# Oleoylethanolamide, Neuroinflammation, and Alcohol Abuse

**DOI:** 10.3389/fnmol.2018.00490

**Published:** 2019-01-09

**Authors:** Laura Orio, Francisco Alen, Francisco Javier Pavón, Antonia Serrano, Borja García-Bueno

**Affiliations:** ^1^Department of Psychobiology and Methods in Behavioral Science, Faculty of Psychology, Complutense University of Madrid, Madrid, Spain; ^2^Red de Trastornos Adictivos (RTA), Instituto de Salud Carlos III (ISCIII), Madrid, Spain; ^3^Unidad de Gestión Clínica de Salud Mental, Instituto de Investigación Biomédica de Málaga (IBIMA), Hospital Regional Universitario de Málaga–Universidad de Málaga, Málaga, Spain; ^4^Department of Pharmacology and Toxicology, Faculty of Medicine, Complutense University of Madrid, Madrid, Spain; ^5^Centro de Investigación Biomédica en Red de Salud Mental, IMAS and IUING, Madrid, Spain

**Keywords:** OEA oleoylethanolamide, alcohol, neuroinflammation, lipids, acylethanolamides, drugs of abuse, inflammation, neuroprotection

## Abstract

Neuroinflammation is a complex process involved in the physiopathology of many central nervous system diseases, including addiction. Alcohol abuse is characterized by induction of peripheral inflammation and neuroinflammation, which hallmark is the activation of innate immunity toll-like receptors 4 (TLR4). In the last years, lipid transmitters have generated attention as modulators of parts of the addictive process. Specifically, the bioactive lipid oleoylethanolamide (OEA), which is an endogenous acylethanolamide, has shown a beneficial profile for alcohol abuse. Preclinical studies have shown that OEA is a potent anti-inflammatory and antioxidant compound that exerts neuroprotective effects in alcohol abuse. Exogenous administration of OEA blocks the alcohol-induced TLR4-mediated pro-inflammatory cascade, reducing the release of proinflammatory cytokines and chemokines, oxidative and nitrosative stress, and ultimately, preventing the neural damage in frontal cortex of rodents. The mechanisms of action of OEA are discussed in this review, including a protective action in the intestinal barrier. Additionally, OEA blocks cue-induced reinstatement of alcohol-seeking behavior and reduces the severity of withdrawal symptoms in animals, together with the modulation of alcohol-induced depression-like behavior and other negative motivational states associated with the abstinence, such as the anhedonia. Finally, exposure to alcohol induces OEA release in blood and brain of rodents. Clinical evidences will be highlighted, including the OEA release and the correlation of plasma OEA levels with TLR4-dependent peripheral inflammatory markers in alcohol abusers. In base of these evidences we hypothesize that the endogenous release of OEA could be a homeostatic signal to counteract the toxic action of alcohol and we propose the exploration of OEA-based pharmacotherapies to treat alcohol-use disorders.

## Introduction: Conceptualization of Neuroinflammation and Its Relationships With Neuropsychiatric Disorders

### Framework for Neuroinflammation in CNS Pathologies

Immunopsychiatry is a renewed biomedical discipline in continuous expansion that studies the interaction/s between the brain and the immune system. It is aimed to find new molecular/cellular targets for the development of original therapies for the treatment of psychiatric disorders (Pariante, 2015; Khandaker et al., 2017). It is based on increasing evidences from both preclinical and clinical studies supporting the existence of a “low grade” neuroinflammatory response with relevance in the etiophysiopathology of psychiatric disorders [recently reviewed in Bauer and Teixeira (2018)]. In particular cases such as schizophrenia and other psychotic diseases, this neuroinflammation has been demonstrated by means of brain *postmortem* evidence and imaging techniques, although some authors have alerted about the need for more selective markers of glial activation and the combination with diverse methodologies at molecular/cellular level (Trepanier et al., 2016; Notter et al., 2018). Positron emission tomography (PET) imaging and *postmortem* evidences are also extended to major depression, bipolar disorder, autism spectrum disorder, and drug abuse, but also with some degrees of controversy (van der Doef et al., 2015; Mechawar and Savitz, 2016; Kim and Won, 2017).

Neuroinflammation is defined as the homeostatic response of the central nervous system (CNS) against injury. It presents shared characteristics with peripheral inflammation but also retains specific features, such as the existence of the brain–blood barrier (BBB) and the cerebrospinal fluid, or the activation of glial cells. Neuroinflammation is a hot topic in the study of CNS pathologies and, recently, some authors have tried to redefine and to delimitate the concept, paying special attention to the whole process different roles (homeostatic vs. detrimental), triggers, and degrees of severity (DiSabato et al., 2016). One fundamental consideration is that classical inflammatory-related cells or mediators, such as microglia or cytokines, can regulate physiological processes in the CNS not always strictly related to neuroinflammation (Estes and McAllister, 2014).

One consensus idea is that the neuroinflammatory response is a necessary process for coping with any type of injury (Grippo and Scotti, 2013), but may become deleterious in severe, non-controllable, and/or long-lasting conditions (Black, 2002).

In particular, the concomitant and/or exclusive overproduction and activity of pro-inflammatory mediators such as cytokines [tumor necrosis factor alpha (TNF-α), interleukin 1beta (IL-1β), and IL-6], chemokines [monocyte chemoattractant protein 1 (MCP-1/CCL2) and fractalkine (CX3CL1)], prostaglandins [prostaglandin E_2_ (PGE2)], kinases [extracellular signal-regulated kinase, c-Jun N-terminal kinase, mitogen-activated protein kinase (MAPK)], pattern recognition receptors (PRR) [toll-like receptors (TLR)], nuclear factors [nuclear factor kappa B (NF-κB) and activator protein 1 (AP-1)], signal transducer and activator of transcription (STAT), and enzymes [cyclooxygenases (COX), inducible NO synthase (iNOS)] could lead to alterations in the normal structure and function of the CNS.

Thus, the neuroinflammatory response has been related to neurodegeneration by oxidative/nitrosative stress, neurodevelopment, neuroplasticity and cellular metabolism, survival, and differentiation (Okun et al., 2011; Leza et al., 2015; Wohleb et al., 2016; De Picker et al., 2017). At the end, all these alterations can even affect other multifaceted processes, such as monoaminergic and glutamatergic neurotransmission (Ogyu et al., 2018), neuroendocrine responses (Pariante, 2017), cognition (Skaper et al., 2014), and ultimately behavior (Dantzer et al., 2008; McCusker and Kelley, 2013).

### Regulation of Neuroinflammation and Trigger Mechanisms

The neuroinflammatory response must be finely regulated to avoid its deleterious consequences. In response to immune activation endogenous anti-inflammatory and antioxidant mechanisms are activated in the CNS. There are three mechanisms that are currently receiving especially attention: (1) Synthesis of the cyclopentenone prostaglandin 15-deoxy-PGJ_2_ (15d-PGJ_2_) (Prasad et al., 2008). This is the proposed endogenous ligand for the gamma isoform of peroxisome proliferator-activated nuclear receptors (PPARγ), a transcription factor whose main effect is to mitigate inflammation by repressing NF-κB signaling (Garcia-Bueno et al., 2008). PPARγ may be pharmacologically activated by the antidiabetic drugs thiazolidinediones, which exert anti-inflammatory, anti-excitotoxic, and pro-energetic effects in the brain (Garcia-Bueno et al., 2008); (2) The activation of the antioxidant pathway orchestrated by the nuclear transcription factor (erythroid-derived 2)-like 2 (NRF2). In presence of oxidative stress signals, NRF2 translocates into the nucleus where it binds to consensus sequences of antioxidants response elements (ARE). ARE encode a wide variety of antioxidant enzymes including some dedicated to glutathione synthesis and to the elimination of oxygen reactive species (Zhang et al., 2013); and (3) polarization of microglia to the anti-inflammatory phenotype M2. M2 microglia secretes anti-inflammatory cytokines such as the transforming growth factor beta (TGFβ) and IL10 (Hu et al., 2014). Increased M2 profile could provide neuroprotection under CNS pathological conditions, and its modulation is emerging as a promising target in neuropsychiatric diseases (Reus et al., 2015).

Another important neuroinflammation-related topic to explain whether immune activation is a neuropsychiatric etiological factor or just an epiphenomenon is the study of its triggering mechanism/s. The nature and extent of the immune activation depend on whether it is caused by endogenous or exogenous factors, but the physiopathological consequences appear to be directly related to the precise moment of this activation. This area of research is especially focused on the study of the innate immune TLRs family. TLRs belong to the super-family of PRRs that detect conserved pathogen-associated molecular patterns (PAMPs) (Akira and Takeda, 2004). The canonical example of PAMPs is the endotoxin lipopolysaccharide (LPS) (Phelps et al.) from the outer membrane of Gram-negative bacteria (Poltorak et al., 1998a,b). LPS is recognized by TLR4, the first member of the family described in humans (Medzhitov et al., 1997).

Toll-like receptors also recognize multiple endogenous ligands, released as cellular or tissue danger/damage signals, named damage-associated molecular patterns (DAMPs) or alarmins (Seong and Matzinger, 2004). In the case of TLR4, the best described DAMPs are fibrinogen, heat shock proteins 60–70 (HSP60-70), and high-mobility-group box protein 1 (HMGB-1) (Piccinini and Midwood, 2010). The implication of DAMPs in the process called “sterile inflammation” supports the view of TLR4 as a ubiquitous sentinel receptor and some authors have remarked the importance of such subtype of neuroinflammation in psychiatric disorders (Ratajczak et al., 2018). Indeed, in the last years, some evidence suggests a role of TLR4 in stress and stress-related neuropsychiatric diseases (Garcia Bueno et al., 2016). There are several mechanisms related to TLRs activation in neuropsychiatric diseases that deserve further investigation: (1) bacterial translocation of the Gram-negative enterobacteria (“leaky gut”). Increased intestinal barrier permeability and bacterial translocation have been reported in patients with major psychiatric disorders. Patients also present an altered microbiome and, in some cases, intestinal inflammation (Kelly et al., 2015); (2) prenatal infection (bacteria, virus, and protozoa) induced maternal immune activation (MIA)/inflammation and the resultant oxidative/nitrosative stress. In this way, an infection during the gestational period is a risk factor for developing psychiatric disease in the progeny over the course of a lifetime (Estes and McAllister, 2016). This idea of early immune-dependent alterations in the neurodevelopment supports the use of animal models based on MIA and other prenatal stressors exposure for the study of psychiatric disease (Markham and Koenig, 2011; Meyer, 2014); and (3) other possible sources of activation of the innate immune system have been recently suggested for major depressive disorder and could be extended to other psychiatric diseases. These include psychosocial stressors, physical inactivity, obesity, smoking, poor dental care or overall hygiene, and sleep deprivation (Berk et al., 2013).

### Approaches to Study Neuroinflammatory-Based Conditions

The study of the inflammatory response in psychiatric disease is actively focusing on the search of new diagnostics to monitor the natural course of the disease (trait or state biomarkers, risk/protection factors) (Lopresti et al., 2014; Tomasik et al., 2016; Sayana et al., 2017). However, finding a unique *golden marker* for the different neuropsychiatric pathologies is greatly difficult and challenging due to the heterogeneity of complex mental disorders, the unspecific nature of the inflammatory response, and the great number of confounding factors that trigger immune alterations (psychological stress exposure, treatment, metabolic and other co-morbid conditions, diet, age, latent infections, use and abuse of psychoactive drugs, etc.). The most logical approach is to study all the elements of an inflammatory pathway, including the regulatory mechanisms and their relationship with symptomatology, through the use of synergic methodology, such as fMRI, brain morphometry, *postmortem* studies, cognitive assessments, peripheral biomarkers, and genetic/epigenetic studies (Leza et al., 2015).

In spite of the existent gaps in the knowledge of the precise role of neuroinflammation in the etiophysiopathology of neuropsychiatric disorders, there is an increasing number of clinical trials evaluating the use of classical anti-inflammatory/antioxidant agents as a co-adjuvant strategy to current pharmacological treatments. Some of these agents are nonsteroidal anti-inflammatory drugs (NSAIDs), selective COX-2 inhibitors (COXIBs), cytokine antagonists, omega-3 polyunsaturated fatty acids, prebiotics, probiotics, minocycline, and *N*-acetylcysteine (Keller et al., 2013; Rosenblat et al., 2016; Husain et al., 2017). In general, only modest clinical effects have been reported with compounds capable of blocking different pro-inflammatory pathways, suggesting the need to explore new compounds and combined strategies. Specifically, the change of paradigm toward the use of compounds that enhance the existent endogenous anti-inflammatory mechanisms, such as the activation of PPAR isoforms that function as transcription factors, has gained special attention in the last years (Garcia-Bueno et al., 2010; Rolland et al., 2013; Colle et al., 2017).

In this sense, this review introduces the role of endogenous compounds such as the acylethanolamides as anti-inflammatory agents and their potential to treat neuropsychiatric disorders. Within the neuropsychiatric disorders, here we focus on the alcoholic pathology and, specifically, we revise the exiting evidence of the compound oleoylethanolamide (OEA) to modulate alcohol abuse-induced neuroinflammation.

## Alcohol Abuse and Neuroinflammation

### Alcohol-Use Disorder as a CNS Pathology With a Neuroinflammatory Component

According to the World Health Organization and the National Institute on Drug Abuse (NIDA), addiction is conceptualized as a chronic and recurrent disease of the CNS. Repeated use and abuse of drugs may induce an addiction disorder and, among all drugs of abuse, alcohol is one of the most widely used psychoactive drugs worldwide, whose consumption originates major public health problems in our society, including addiction. Although many individuals regularly consume alcohol in moderate doses with no significant adverse consequence, a substantial number drink alcohol in abuse patterns, such as binge drinking. Binge drinking is a common abuse pattern among adolescents and young adults, and it is defined as a short-term pattern of consumption (four or five drinks in about 2 h) that increases the blood alcohol concentration above 80 mg/dl (National Institute on Alcohol Abuse and Alcoholism [NIAAA], 2004). Chronic alcohol consumption, especially in binge drinking episodes, contributes to several medical complications as well as to a higher risk of developing alcohol-use disorders (AUDs).

Alcohol binge drinking and chronic alcohol consumption are associated with the induction of neuroinflammation (Obernier et al., 2002, XX), and growing body of evidence suggests that this neuroinflammatory process may contribute to alcohol-induced brain damage and neurodegeneration (Valles et al., 2004; Hamelink et al., 2005; Crews et al., 2006; Tajuddin et al., 2014). Moreover, this inflammation-induced damage caused by alcohol abuse is associated with the development of cognitive and behavioral impairments in preclinical studies (Pascual et al., 2011). In humans, blood peripheral inflammatory markers have been associated with alcohol abuse, specifically bingeing or intoxication (Orio et al., 2017; Pascual et al., 2017) and neuroinflammatory components have been detected in human postmortem brains of alcoholics (Rubio-Araiz et al., 2017).

### Role of TLR4 in Alcohol-Induced Neuroinflammation

Toll-like receptors are a family of receptors involved in the regulation of innate immune responses to infections and CNS injury (Jin et al., 2008; Okun et al., 2009) comprising 10 members in humans and 12 in mice (Brubaker et al., 2015). Among them, TLR2, TLR3, and TLR4 are up-regulated in the frontal cortex of *postmortem* AUD patients as well as in the cortex of mice chronically treated with alcohol (Crews et al., 2013). Alcohol also induces the expression of TLR7 in the hippocampus of *postmortem* AUD patients and in rat brain slice culture after alcohol treatment (Coleman et al., 2017a). TLR4 is one of the innate immune receptors receiving more attention in the context of neuroinflammation-associated neuropsychiatric disorders. Growing evidence suggests that the activation of TLR4 signaling plays an important role in the pathogenesis of alcohol-induced inflammatory brain damage. Thus, it has been shown that alcohol activates TLR4 response in brain, astrocytes, microglia, and macrophages (Blanco et al., 2005; Fernandez-Lizarbe et al., 2008, 2009; Qin et al., 2008; Alfonso-Loeches et al., 2010). Moreover, it has been reported the absence of inflammatory response in microglia of TLR4-deficient mice after alcohol treatment (Fernandez-Lizarbe et al., 2009). Similarly, the knockdown of the expression of TLR4 inhibits the production of pro-inflammatory mediators by blocking the activation of MAPK and NF-κB pathways in astrocytes (Alfonso-Loeches et al., 2010). The activation of TLR4 also plays an important role in the behavioral and cognitive dysfunctions associated with alcohol-induced neuroinflammation damage since these deficits, such as memory impairments and anxiety-like behaviors, are not observed in TLR4-deficient mice after alcohol treatment (Pascual et al., 2011).

### Alcohol-Induced HMGB1 Activates TLR4

Damage-associated molecular patterns are endogenous agonists of TLRs that are involved in numerous non-infectious neurological disorders, including AUDs (Crews et al., 2017). Among these endogenous TLR4 agonists, HMGB1 is a pro-inflammatory signal that plays a key role in AUDs. In rats, alcohol exposure during adolescence activates HMGB1/TLR4 danger signaling in the brain, and this activation persists into adulthood correlating with the presence of cognitive deficits (Vetreno and Crews, 2012). Other studies in rodents have shown that chronic alcohol treatment increases HMGB1 in cortex and cerebellum (Crews et al., 2013; Lippai et al., 2013), and cortical HMGB1 is also upregulated after alcohol binges (Antón et al., 2017a). Additionally, young female regular alcohol binge drinkers show elevated HMGB1 in plasma (Antón et al., 2017b) and the expression of HMGB1 is increased in the frontal cortex of *postmortem* AUD patients (Crews et al., 2013). The activation of HMGB1 by alcohol causes its translocation from the nucleus, and then, it is released from neurons to the extracellular space triggering alcohol-induced neuroimmune signaling through direct binding to both glial and neuronal TLR4 (Zou and Crews, 2014). The activation of microglial TLR4 by HMGB1 also induces the expression and release of more HMGB1 from neurons, which magnifies the inflammatory response (Zou and Crews, 2014).

In addition to TLR4 signaling pathway, HMGB1 can also bind and activate other targets, such as other TLRs and the receptor for advanced glycation end products (RAGE) (Park et al., 2004; Volz et al., 2010). The expression of RAGE is up-regulated in the *postmortem* brain of AUD patients and correlates with an early age of drinking (Vetreno et al., 2013). Increased brain HMGB1 levels have been associated with cognitive deficits, effects that are mediated through both TLR4 and RAGE receptors (Mazarati et al., 2011). A recent study reports that HMGB1/IL-1β heterodimer contributes to the immune activation induced by alcohol (Coleman et al., 2017b). In fact, an increase of HMGB1/IL-1β complexes was found in the hippocampus of mice after an acute binge alcohol treatment and in *postmortem* tissue of AUD patients (Coleman et al., 2017b).

### NF-κB Canonical Pro-inflammatory Signaling Pathway

Toll-like receptors belong to a large superfamily that includes the interleukin-1 receptors (IL-1Rs) (Akira and Takeda, 2004), and the activation of these receptors contributes to the neuroinflammation process (Chen et al., 2007). The activation of IL-1Rs and TLRs induces, via myeloid differentiation factor 88 (Myd88)-dependent pathway, the recruitment of downstream signaling molecules, including IL-1R-associated kinase (Miyake et al., 2015), tumor necrosis factor receptor-associated factor 6 (TRAF6), and the NF-κB-inducing kinase (Akira and Takeda, 2004). The recruitment of these molecules triggers the stimulation of signaling pathways, including the MAPK pathway, and the activation of transcriptional factors, such as the NF-κB and the AP-1, which lead to the induction of genes encoding inflammation-associated molecules and cytokines (Akira and Takeda, 2004).

**FIGURE 1:**
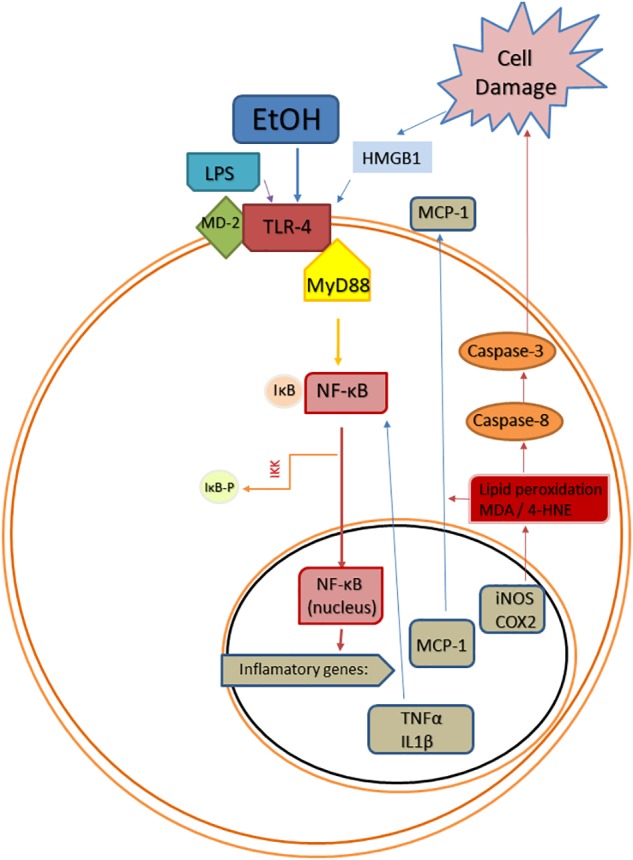
Scheme of convergent Toll-like receptor 4 (TLR-4) activating pathways after ethanol (EtOH) exposure. EtOH is a direct agonist of TLR4 and can also activate it through the induction of PAMPs, such as bacterial LPS (Phelps et al.), or DAMPs, such as high mobility group box 1 protein (HMGB1). Abbreviations: MD-2, lymphocyte antigen 96; MyD88, myeloid differentiation primary response 88; NF-κB, nuclear factor kappa B; IκB, inhibitor of κB; IKK, inhibitor κB kinase; IKK-p, phosphorylated inhibitor κB kinase; TNF-α, tumor necrosis factor alpha; IL1β, interleukin 1beta; MCP-1, monocyte chemoattractant protein-1; iNOS, inducible NO synthase; COX-2, cyclooxygenase 2; MDA, malondialdehyde; 4-HNE, 4-hydroxynonenal.

The transcription factor NF-κB plays a key role in alcohol-induced neuroinflammation. Alcohol activates NF-κB in the rat brain (Ward et al., 1996), and *in vivo* and *in vitro* studies have shown that alcohol increases the NF-κB-DNA binding (Crews et al., 2006; Zou and Crews, 2006, 2010), inducing the transcription of multiple pro-inflammatory genes, such as IL-1β, TNF-α, MCP-1, COX-2, or iNOS (Valles et al., 2004; Blanco et al., 2005; Figure 1). The induction of COX-2 by alcohol involves a TLR4-mediated signaling, since chronic alcohol-induced up-regulation of COX-2 is not observed in TLR4 knockout mice (Alfonso-Loeches et al., 2010), and alcohol-induced COX-2 and iNOS upregulation has been associated to cell death (Blanco et al., 2005; Antón et al., 2017a).

Additionally, COX-2 and iNOS play a key role in the alcohol-induced inflammatory brain damage during adolescence (Pascual et al., 2007). Additionally, the expression of NF-κB is associated to alcohol preference in rodents (Mulligan et al., 2006), while in humans, a variation in NF-κB1 is linked to increased risk of AUD (Edenberg et al., 2008).

### Role of Oxidative Stress in Alcohol-Induced Neuroinflammation

In addition to the NF-κB-signaling pathway, another innate immune gene activated by alcohol is the nicotinamide adenine dinucleotide phosphate (NADPH) oxidase, an enzyme that produces reactive oxygen species (ROS), inducing oxidative stress and contributing to cell damage (Qin et al., 2008; Qin and Crews, 2012). The activation of this enzyme is involved in the pathology of neurodegenerative diseases, such as Alzheimer’s disease and Parkinson’s disease (Shimohama et al., 2000; Wu et al., 2003; Ma et al., 2017). Previous studies have found an activation of NADPH oxidase and an increase in the production of ROS in the orbitofrontal cortex of *postmortem* AUD patients as well as in mice after a chronic alcohol treatment (Qin and Crews, 2012). The production of ROS is also associated with the activation of NF-κB transcription, contributing to an amplification of the pro-inflammatory cascade (Crews and Vetreno, 2016). However, the administration of the antioxidant butylated hydroxytoluene reduces the NF-κB-DNA binding induced by alcohol, blocking the activation of this transcription factor and preventing the neuroinflammation (Crews et al., 2006; Zou and Crews, 2010). Several studies from Tajuddin et al. (2013, 2014) have described the alteration of pro-oxidative pathways associated to neuroinflammation after binge alcohol treatment, and these affects are prevented by docosahexaenoic acid. These pathways include the protein aquaporin-4 (AQP4) water channel as well as the neuroinflammation-linked enzymes: key phospholipase A2 (PLA2) family members and poly (ADP-ribose) polymerase 1 (PARP-1). AQP4 is expressed mainly in astroglia and is involved in the regulation of cellular brain edema, playing a key role in the neuroinflammation process (Saadoun and Papadopoulos, 2010; Fukuda and Badaut, 2012). Previous *in vitro* studies have shown that repetitive alcohol intoxication bouts increase AQP4 levels (Sripathirathan et al., 2009). Therefore, oxidative stress is involved in alcohol-induced brain damage, and the use of drugs with antioxidant effects may be an effective treatment against the neuroinflammation and neurodegeneration induced by alcohol.

## Oleoylethanolamide Is a Member of the Anti-Inflammatory and Neuroprotectant Acylethanolamide Family

**FIGURE 2:**
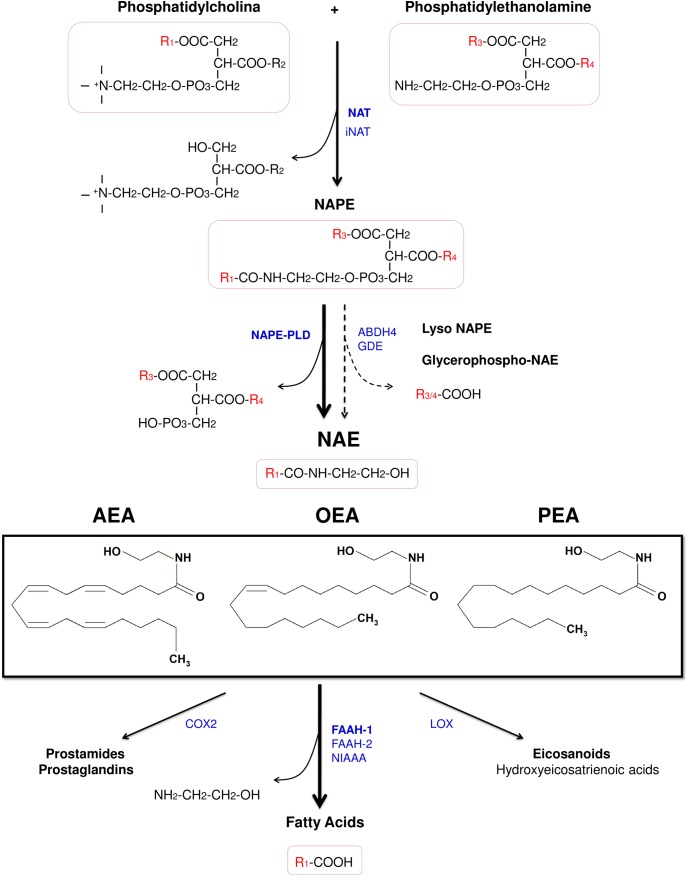
Chemical structure of the acylethanolamides anandamide (AEA), oleoylethanolamide (OEA), and palmitoylethanolamide (PEA), and pathways for their synthesis, degradation, and oxidation. Abbreviations: NAT, Ca^2+^-dependent *N*-acyltransferase; iNAT, Ca^2+^-independent NAT; NAPE, *N*-acylphosphatidylethanolamine; NAPE-PLD, NAPE-hydrolyzing phospholipase D; ABDH4, α/β-hydrolase domain-containing protein 4; GDE, glycerophosphodiesterase; NAE, acylethanolamide (*N*-acylethanolamine); AEA, anandamide (*N*-arachidonoylethanolamine); OEA, oleoylethanolamide (*N*-oleoylethanolamine); PEA, palmitoylethanolamide (*N*-palmitoylethanolamine); COX-2, cyclooxygenase-2; FAAH, fatty acid amide hydrolase; NIAAA, *N*-acylethanolamine-hydrolyzing acid amidase; LOX, lipoxygenase.

The acylethanolamides (also known as *N*-acylethanolamines) are a class of naturally occurring lipid mediators involved in the regulation of a great deal of homeostatic functions (Orio et al., 2013). The endocannabinoid AEA is structurally an acylethanolamide, and other bioactive acylethanolamides are the anorexic lipid mediator OEA (Rodriguez de Fonseca et al., 2001) and the anti-nociceptive mediator palmitoylethanolamide (PEA) (Calignano et al., 2001; Figure 2). In this review, we will summarize the anti-inflammatory and neuroprotectant actions of these three acylethanolamides and we will focus on the specific role of OEA in neuroinflammation and alcohol abuse.

### Synthesis, Degradation, and Receptor Binding of Acylethanolamides

Despite of the fact that acylethanolamides are widely divergent in function, they share common synthesis and inactivation pathways (Fonseca et al., 2013; Figure 2). Acylethanolamides are composed of a fatty acid and ethanolamine. Fatty acids can be synthesized within the brain, but polyunsaturated fatty acids are mainly supplied by the blood. From the diet, some fatty acids are incorporated to the blood as precursors of other polyunsaturated fatty acids such arachidonic acid (AA) (Bazinet and Laye, 2014). Many of these fatty acids are largely esterified to the cell membrane phospholipid and they participate in signal transduction directly or, after enzymatic conversion, they are transformed in a variety of lipid mediators. Thus, these bioactive lipid derivatives regulate numerous processes within the CNS, including neurotransmission, cell survival, and neuroinflammation (Bazinet and Laye, 2014). Otherwise, fatty acids can also influence brain function through modulation of the endocannabinoid system, a signaling system with a broad spectrum of biological functions.

Acylethanolamides are synthesized by sequential catalyses following a classical “transacylation-phosphodiesterase pathway” from glycophospholipids. Primarily, a cAMP and Ca^2+^-dependent *N*-acyltransferase (NAT) generates *N*-acylphosphatidylethanolamine (NAPE) (Cadas et al., 1997) and, subsequently, a non-selective NAPE-hydrolyzing phospholipase D (NAPE-PLD) releases acylethanolamide together with a phosphatidic acid molecule (Okamoto et al., 2004). However, there are secondary pathways to this canonical biosynthesis, for example through a double *O*-deacylation of NAPE to generate NAE by the enzyme α/β-hydrolase domain-containing protein 4 (ABDH4) (Simon and Cravatt, 2006; Ueda et al., 2013; Figure 2).

Regarding the degradation, acylethanolamides are rapidly inactivated by a process that includes reuptake and hydrolysis, although only the hydrolytic step has been well-characterized. The hydrolysis of acylethanolamide to free fatty acid and ethanolamine is a major pathway that is mainly performed by a membrane-bound fatty acid amide hydrolase (FAAH), which is localized in the endoplasmic reticulum (Cravatt et al., 1996). In addition to the original FAAH (designated FAAH-1), an isozyme of FAAH (FAAH-2) has been identified in human lipid droplets but not in rodents (Wei et al., 2006). Other enzymes such as COX-2 and lipoxygenases, which participates in inflammatory functions, are able to inactivate these acylethanolamides by oxygenation (Ueda et al., 2013; Figure 2). Nevertheless, a growing body of evidence has demonstrated the existence of alternative enzymes and pathways for acylethanolamide metabolism, such as Ca^2+^-independent NAT enzymes [(members of the H-RAS-like suppressor subfamily (HRASLS) and phospholipase A/acyltransferase (PLA/AT)], NAPE-PLD independent multistep pathways via *N*-acylated lysophospholipid, and the hydrolytic enzyme *N*-acylethanolamine-hydrolyzing acid amidase (NAAA) that preferentially hydrolyzes PEA (Ueda et al., 2013).

Unlike AEA, the other acylethanolamides OEA and PEA do not bind to cannabinoid receptors, despite PEA was initially described as a potential CB_2_ receptor agonist. The biological functions of these acylethanolamides appear to be mainly mediated by activating PPARα (Fu et al., 2003; LoVerme et al., 2005), although PEA has lower potency as agonist than OEA. However, the presence of other acylethanolamides has been known to enhance AEA activity at both cannabinoid and vanilloid receptors by an “*entourage effect*” (Smart et al., 2002; Ho et al., 2008). Interestingly, PEA activity at PPARα is efficacious to induce antinociceptive effects but not to induce satiety (LoVerme et al., 2006), whereas OEA is more potent as PPARα activator and exerts weaker antinociceptive effects than PEA (Suardiaz et al., 2007).

In addition to PPARα, acylethanolamides can modulate other targets such as PPARγ or PPARδ isoforms (Fu et al., 2003; Paterniti et al., 2013), other non-cannabinoid GPCRs (e.g., GPR55 and GPR119) (Overton et al., 2006; Godlewski et al., 2009), or the transient receptor potential vanilloid type-1 (TRPV-1) (Van Der Stelt and Di Marzo, 2004; Almasi et al., 2008). It is to note that there are physiological actions induced by acylethanolamides that are not explained by a modulation of these targets previously commented and, consequently, new targets should be possibly elucidated in the next few years.

### Anti-inflammatory and Neuroprotective Actions of Acylethanolamides

As mentioned in the section “Introduction: conceptualization of neuroinflammation and its relationships with neuropsychiatric disorders,” chronic neuroinflammation is a common characteristic of a broad range of dysfunctions including psychiatric disorders, neurodegenerative disorders, ischemia, hypoxia, trauma, brain aging and infection, and, for our purposes, even addictive disorders, which result in brain damage [for review see Skaper et al. (2013) and Herrera et al. (2016)]. The following paragraphs refer to the evidence in favor of an anti-inflammatory and neuroprotective role of acylethanolamides in such brain disorders.

Emerging evidence supports the relevance of acylethanolamides in neuroinflammation. Several studies have shown that stimulated glial cells produce these lipid mediators (Walter et al., 2002; Muccioli and Stella, 2008; Skaper et al., 2013). The endogenous levels of acylethanolamides are upregulated in several CNS pathologies, such as multiple sclerosis (Baker et al., 2001), stoke (Schabitz et al., 2002), ischemia (Degn et al., 2007), Huntington’s disease (Bisogno et al., 2008), stress and depression (Hill et al., 2009), post-traumatic stress disorder (Hauer et al., 2013), Parkinson’s disease (Gonzalez-Aparicio and Moratalla, 2014), etc.

A recent study demonstrated that the pharmacological inhibition of FAAH and subsequent increase of AEA, PEA, and OEA in the rat brain produce an attenuation in the expression of cytokines and glial mediators following a systemic administration of LPS (Henry et al., 2017). This anti-inflammatory actions of the acylethanolamides are associated with reduction in anhedonia, although they didn’t block LPS-induced acute sickness behavior (Henry et al., 2017). Interestingly, a gradient release of AEA, PEA, and OEA was found by cerebral microdialysis in a patient suffering with hemispheric stroke, with less concentration of AEA and higher concentrations of OEA in the lesioned area, highlighting the neuroprotective homeostatic role of OEA against the other acylethanolamides (Schabitz et al., 2002).

Finally, it is to note that the neuroprotective actions of acylethanolamides could not be restricted to their brain release, since they rapidly cross the BBB after systemic administration (Artamonov et al., 2005; Gonzalez-Aparicio et al., 2014). In this regard, the exogenous peripheral administration of PEA and OEA exerts neuroprotective effects in several models of brain injury and neurological disorders [for review see Herrera et al. (2016)].

### Target Receptors for Acylethanolamide-Induced Anti-inflammatory Actions

Acylethanolamides may exert neuroprotective actions by several mechanisms involving both receptor activation and interaction with other signaling pathways related to the regulation of neuroinflammation. Among these signaling pathways, TLRs have emerged as central keys in the innate immune and neuroimmune responses. As mentioned before, TLRs are activated by PAMPs and they are highly expressed in glial cells (Jack et al., 2005). Their activation lead to intracellular signaling cascades involving several kinases [e.g., MAPKs and tumor growth factor-β-activated kinase 1 (TAK1)] that converge in the transcription of pro-inflammatory genes by activating the NF-κB and AP-1 (Akira and Takeda, 2004). LPS acts as the prototypical endotoxin because it binds to TLR4 as agonist and promotes an inflammatory response through NF-κB activation (McCoy, 2016). Acylethanolamides may directly interfere with the TLR4 signal transduction (i.e., NF-κB), but also may act indirectly suppressing the production of pro-inflammatory cytokines through multiple mechanisms such as the induction of apoptosis, as an immunosuppressive mode of action, or the induction of inhibitory signals [e.g., anti-inflammatory cytokines (IL-10) and IL-1 receptor antagonist] [for review see McCoy (2016)].

In addition to TLRs, cannabinoid and non-cannabinoid receptors for acylethanolamides are also expressed in glial cells, and their ligands have been shown to negatively modulate TLR-induced neuroinflammation in these cells (Saito et al., 2012). There is evidence that activation of immune cells by inflammatory stimuli modulates the expression of both subtypes of cannabinoid receptors, a fact that has been associated with immune-regulatory effects of cannabinoids (Saito et al., 2012). Several studies have reported that endocannabinoids inhibit the formation of pro-inflammatory cytokines in both human cell cultures and animal models, but the interplay between the signaling pathways of the cannabinoid receptors and TLRs needs to be considered for a better comprehension.

The anti-inflammatory actions of AEA can be related to its ability to inhibit 2-AG metabolism and effects (Maccarrone et al., 2008), since 2-AG signaling appears to be the primary responsible for maintaining integrity and homeostasis of brain function (Xu and Chen, 2015). Microglial cells express high levels of CB2 receptor in patients with neurological disorders (e.g., multiple sclerosis and Alzheimer’s disease) (Benito et al., 2003; Yiangou et al., 2006). Indeed, a recent study showed that AEA attenuates LPS-induced neuroinflammation (based on NO production) in microglial cultures via CB_2_ receptors (Malek et al., 2015). Thus, while the administration of a CB_2_ receptor antagonist partially prevented the neuroprotective effects of AEA on microglia, CB_1_ or GPR55 antagonists have no effects. Nevertheless, CB_1_ receptors also participate in neuroinflammation regulation. For example, AEA inhibits the expression of endothelial receptors related to leukocyte transmigration in multiple sclerosis through CB_1_ receptor (Greenwood et al., 2011). Furthermore, the activation of CB_1_ receptors modulates stress-induced conditions and neuroinflammation through several mechanisms, including the inhibition of inflammatory response genes via NF-κB and enzymes such as inducible iNOS and COX-2 (Zoppi et al., 2011).

The activation of PPARα and other isoforms modulates oxidative stress, neurotransmission, neurogenesis, glial cell proliferation/differentiation, and neuroinflammation (Fidaleo et al., 2014) through negative transcriptional regulation of inflammatory response genes. A study in rats from our lab showed that OEA and PEA prevent LPS-induced NF-κB activation, the expression of iNOS and COX-2, accumulation of NO, and lipid peroxidation in the frontal cortex (Sayd et al., 2015). However, to date, it is unproven that this brain anti-inflammatory effects are directly mediated or not by PPARα receptors. PEA has been proven to exert anti-inflammatory effects in ischemia through an intracellular mechanism independent of known acylethanolamides receptors (Garg et al., 2010).

Finally, TRPV-1 appears to play a bidirectional role in the modulation of inflammatory cytokines. TRPV-1 has been reported to be functionally expressed in rodent and human glial cells (Martins et al., 2014). TRPV-1 activation promotes Na^+^ and Ca^2+^ influx and membrane depolarization, and directly affects cellular responses such as proliferation, migration, release of inflammatory cytokines, and oxidative stress. Several studies show that TRPV-1 regulates synaptic transmission and plasticity in neurons and interacts with pro-inflammatory cytokines. A growing body of recent literature supports that TRPV-1 activation induces an amplification of pro-inflammatory responses, whereas TRPV-1 inhibition reduces the release of cytokines and neurotoxicity (Huang et al., 2015; Saffarzadeh et al., 2015). Therefore, TRPV-1 activation leads to aggravate the inflammatory response in certain neurological diseases (e.g., experimental autoimmune encephalomyelitis, epilepsy, and brain ischemia) (Kong et al., 2017). In contrast, other studies indicate a protective role of TRPV-1 in neurological diseases. Thus, agonists of TRPV-1 have been shown that act as anti-inflammatory and immunomodulatory agents by inhibiting pro-inflammatory cytokines (e.g., TNF-α, IL-1β, and IL-17) (Tsuji and Seino, 2010). Moreover, TRPV-1 also showed protective roles in vascular dementia and Huntington’s disease by inhibiting oxidative stress (Gupta and Sharma, 2014; Gupta et al., 2014). Additional mechanisms to reduce inflammation by modulating inflammatory signaling pathways have been suggested. For example, vanilloids can inhibit LPS-induced production of NO and regulate protein and gene expression of iNOS and COX-2 (Chen et al., 2003).

A potential explanation of these paradoxical effects is associated with a desensitization of TRPV-1. There is evidence of TRPV-1 sensitization by inflammatory mediators (e.g., cytokines, chemokines, neurotransmitters, lipids, and growth factors) direct or indirectly, inducing strengthen TRPV-1 activity. Thus, several studies have reported the existence of co-expression and functional association between TRPV-1 and receptors of these inflammatory mediators, which can regulate the conductance and trafficking of TRPV-1. Therefore, cytokines can interact with their specific GPCRs, which phosphorylate TRPV-1 and induce changes in intracellular Ca^2+^-dependent signaling pathways such as protein kinases. These changes lead to the downstream activation of transcriptional pathways such as NF-κB and STAT3 (Kong et al., 2017).

All these data suggest that several pro-inflammatory signaling pathways are susceptible to be modulated by acylethanolamides through a direct interaction with receptors such as CB receptors, PPARs and TRPVs, but also through an influence on primary intracellular cascades controlled by TLRs in glial cells, which converge in the regulation of pro-inflammatory genes via transcription pathways such as NF-κB.

## Mechanisms by Which OEA Reduces Alcohol-Induced Neuroinflammation

**FIGURE 3:**
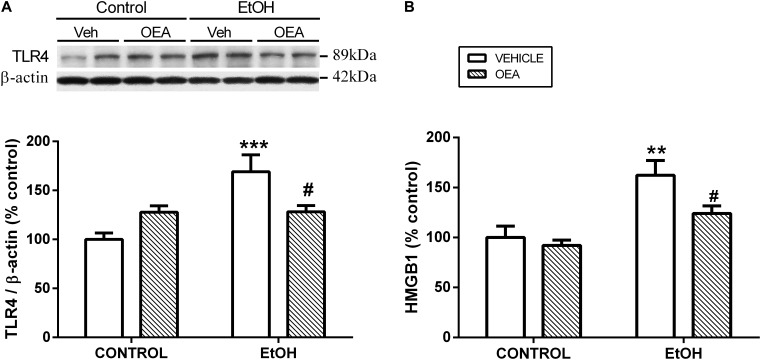
Effects of OEA in cortical TLR4 expression by western blot analyses **(A)** and cortical HMGB1 release by enzyme-linked immunosorbent assay **(B)** in alcohol binge-exposed rats. Modified from Antón et al. (2017a).

Exogenous administration of OEA has proven to exert potent anti-inflammatory, antioxidant, and neuroprotective effects in animals exposed to alcohol binges (Antón et al., 2017a). Specifically, OEA pre-treatment before alcohol binges inhibits alcohol-induced increases in TLR4 expression (Figure 3A) and activation of the NF-κB canonical inflammatory pathway. As a result, OEA inhibits the release of the pro-inflammatory cytokine IL-1β, chemokine MCP-1, and danger molecule HMGB1 (Figure 3B), and the activity of the enzymes COX-2 and iNOS, preventing ethanol-induced lipid peroxidation and apoptotic mechanisms in prefrontal cortex (Antón et al., 2017a; Figure 1). The mechanisms by which OEA reduce innate immunity activation and neuroinflammation induced by alcohol abuse are discussed in the following lines.

### OEA in Alcohol Metabolism

Since ethanol administration induces a rapid release of OEA in the intestine and liver (remember that also in the brain), it could be argued that the satiety factor OEA interferes in the absorption of ethanol. Lower blood ethanol levels would mean decreased ethanol-induced effects, including its action on immune receptors and inflammation. However, the exogenous administration of OEA does not modify blood ethanol levels in animals exposed to alcohol binge intoxication (Antón et al., 2017a), indicative that this is not the protective mechanism of OEA against alcohol actions.

### OEA as a PPARα Agonist

Oleoylethanolamide crosses the BBB reaching the brain rapidly after i.p. administration (Plaza-Zabala et al., 2010; Gonzalez-Aparicio et al., 2014) to exert its actions. The anti-inflammatory actions of OEA could be related to its PPARα agonism within the brain, since activation of this nuclear receptor has been shown to induce changes in inflammatory-related genes by repressing the nuclear factors NF-κB and AP-1 (reviewed in Stahel et al., 2008). However, a direct evidence of the PPARα involvement in OEA-mediated inflammatory actions has not been provided and, indeed, recent reports indicate that endogenous elevations of AEA, OEA, and PEA regulate TLR4-associated neuroinflammation partially through brain TRPV1 but not PPAR receptors (Henry et al., 2017).

### Peripheral Inflammation and Brain Homeostasis

Another plausible mechanism to explain the OEA-induced reduction in neuroinflammation is the anti-inflammatory actions that this biolipid exerts at peripheral level, since the effects of OEA in pro-inflammatory cytokine reduction have been observed both at central and peripheral levels (Antón et al., 2017a). As pointed out in the section “Alcohol Abuse and Neuroinflammation,” systemic immune activation may promote neuroinflammation through different pathways. On the one hand, the sympathetic activity of peripheral pro-inflammatory cytokines activates HPA axis by vagus nerve afferent stimulation and transmits cytokine signals to the brain (Hosoi et al., 2005). On the other hand, peripheral cytokines can reach the brain by the circumventricular organs and/or through saturable cytokine transporters at the BBB, affecting brain homeostasis [reviewed in Dantzer et al. (2008)]. Then, it could be possible that inhibition of alcohol-induced peripheral inflammation by OEA accounts for a reduction in neuroinflammation associated with drug abuse. Specific studies to isolate peripheral from central effects would be needed to ascertain this point.

### Effects in the Blood–Brain Barrier

An increased peripheral inflammatory response could alter the permeability of the BBB (Varatharaj and Galea, 2017), so the ethanol-induced peripheral inflammation could be a risk factor to BBB dysfunction. The main function of the BBB is to protect the CNS from circulating toxins in order to maintain the internal homeostasis. Thus, the infiltration of pathogen molecules or bacterial products from the periphery would make the brain more vulnerable to damage. It has been proven that certain drugs of abuse may alter the structure and functioning of the barrier, including alcohol. Evidence of the BBB disruption and neuroinflammation was shown in *postmortem* alcoholic brains (Rubio-Araiz et al., 2017). The strong anti-inflammatory properties of OEA in the periphery could account for the protective action in the brain, since it could preserve the cytokine-mediated BBB disruption. Alternatively, OEA could have a direct action in the structure of the BBB, protecting alcohol-induced damage in proteins of the basal lamina around the pericytes and endothelial cells. However, a direct action of OEA on the proteins that conform the BBB has not yet been investigated and this hypothesis remains at present speculative.

### Effect in the HPA Axis

It is well known that alcohol modulates the HPA axis, inducing stress-related neuroendocrine adaptations. Acute alcohol activates HPA axis, whereas chronic alcohol consumption may induce tolerance to this effect, blunting the HPA axis over a long-term period (Allen et al., 2011). Glucocorticoids, as final effectors of the HPA axis activation, are important regulators of inflammation, since they are known to exert anti-inflammatory properties by negative regulation of the TLR4/NF-κB pathway, controlling the immunity response. In this regard, an important action of OEA is the reduction of ethanol-induced rise in blood corticosterone levels in rats (Antón et al., 2017a). Elevation in blood corticosterone levels occurs also after experimental simulation of neuroinflammation by systemic administration of LPS, but by contrary, OEA did not revert LPS-induced rise in corticosterone levels (Sayd et al., 2015). These differential effects of OEA on corticosterone levels depending on the cause that induces the rise (ethanol or LPS) are very intriguing and deserve further investigation, but could be related to the different length of OEA administration in each study (subchronic repeated OEA injections in the ethanol study *versus* acute administration in the LPS study). Nevertheless, the inhibition of blood corticosterone levels induced by OEA may be protective for the neurodegeneration induced by ethanol, since elevated circulating corticosterone partially mediates ethanol binge-induced neurotoxicity (Cippitelli et al., 2014).

### Innate Immunity Receptors TLR4 and HMGB1 Release

Among the factors that induce the strong immune activation caused by alcohol abuse are: (1) a TLR4 direct agonism; (2) the upregulation of plasma LPS, which is also a TLR4 agonist; and (3) the overproduction of DAMPS such as HMGB1, which magnifies TLR4 activation (Figure 1). It has been shown that OEA reduces the expression of TLR4 innate immune receptors and its signaling pathway both in the brain and in the gut after ethanol binges (Crews et al., 2013; Antón et al., 2017a, 2018). Regarding the increase of plasma LPS, it is very interesting to note that pretreatment with OEA appears to modulate, at least partially, the alcohol-induced increase in plasma levels of LPS (Antón et al., 2017a), reducing the TLR4 activation and inflammation. Regarding the overproduction of DAMPS, alcohol abuse has been shown to induce the release of HMGB1 in several brain areas in animals and humans, as explained in the section “Alcohol Abuse and Neuroinflammation,” of this review, presumably as a consequence of the TLR4 signaling pathway activation (Antón et al., 2017a,b). This HMGB1 is also a TLR4 agonist and activator of neuroimmune genes, and amplifies the inflammatory response in a vicious cycle. It is to note that young female binge drinkers’ show elevated plasma HMGB1 levels that correlate with circulating levels of OEA. In rats, pretreatment with OEA reduces HMGB1 in cortical samples of alcohol binge exposed animals, which could be the origin or consequence of the reduced TLR4-associated neuroinflammation observed after OEA treatment (Antón et al., 2017a).

### Oxidative Stress

Mechanisms by which ethanol injures the brain include inflammation-derived oxidative stress. In this way, the over-activation of TLR4/NF-κB pro-inflammatory pathway produces oxidative/nitrosative stress, increasing lipid peroxidation in different neuroinflammatory conditions (e.g., stress, LPS, alcohol). This oxidative stress is known to be the consequence of an exacerbated inflammation, but molecules derived from lipid peroxidation and cell damage can also upregulate the inflammatory cycle in a vicious cycle inducing more neuroinflammation. For example, as a consequence of TLR4/NF-κB activation, alcohol abuse induces oxidative stress and redox status-dependent caspase 8 synthesis, which is an upstream intermediate in the caspases’ cascade for apoptosis cell death. In response to damaged tissue, HMGB1 can be released overactivating the proinflammatory TLR4/NF-κB pathway in a vicious cycle, as mentioned before (Figure 1). The anti-oxidant actions of OEA in the rat brain under inflammatory conditions have been shown by us and other authors, since OEA is able to reduce LPS- or alcohol-induced malondialdehyde and/or 4-hydroxynonenal accumulation (lipid peroxidation bioproducts) in rat frontal cortex (Jin et al., 2015; Sayd et al., 2015; Antón et al., 2017a). This antioxidant effect of OEA is accompanied by reductions in caspase-8 and caspase-3 activity and less HMGB1 accumulation, reducing, presumably, the TLR4/NF-κB pathway activation (Antón et al., 2017a).

The antioxidant properties of OEA are multifaceted, as it has been shown in a primary cultured human umbilical vein endothelial cell (HUVEC) H_2_O_2_-induced injury model where OEA acted as scavenger for ROS, as well as increased anti-oxidative enzymes (Ma et al., 2015). More specifically, another *in vitro* study showed that OEA and other *N*-acylethanolamides protected plasma lipoproteins against lipid peroxidation and preserve the activity of the plasma antioxidant enzyme paraoxonase (PON1) from oxidative inactivation (Zolese et al., 2008).

Alcohol abuse activates innate immune genes including the NADPH oxidase, an enzyme that produces ROS, inducing oxidative stress and contributing to cell damage (Qin et al., 2008; Qin and Crews, 2012). As of this writing, a direct implication of OEA in NADPH activity has not been tested yet.

### Protective Actions in the Intestinal Barrier

Alcohol abuse damages the gastrointestinal barrier, allowing the entry of microorganisms and bacterial products, such as LPS, to inner organs and/or the systemic circulation. This phenomenon, called *leaky gut*, has been demonstrated in experimental models of chronic alcohol intake and in alcohol-dependent subjects (Keshavarzian et al., 2009; Leclercq et al., 2012). As a consequence of this *leaky gut* phenomenon, and as we mentioned before, alcohol induces the rise in blood LPS, a component of the wall of Gram-bacteria, which is able to activate TLR4 receptors as an agonist inducing the activation of the inflammatory NF-κB pathway. Alcohol may induce such effect by paracellular or by transepithelial mechanisms. Paracellular mechanisms involve a dysfunction in the tight junction (TJ) proteins of the gut epithelial cells, so the molecules accede the bloodstream through the junctions between cells. Transepithelial mechanisms refer to the pass of substances because of oxidative stress-induced damage or weakness of cell membranes (Bishehsari et al., 2017).

Interestingly, we recently found that OEA pretreatment reduces alcohol-induced elevations in circulant LPS, a reduction that is partial under intraperitoneal (i.p.) treatment (Antón et al., 2017a) and almost total under oral OEA administration (Antón et al., 2018). The origin of such decrease in plasma LPS levels could be a direct action of OEA in the intestinal barrier, modulating gut inflammation and bacteria/bacterial products translocation from the intestinal lumen to the systemic circulation (Antón et al., 2018).

Recent evidence suggests that elevated inflammation in the gut combined with high levels of oxidative/nitrosative stress affect epithelial junctional complexes such as TJ proteins, and contribute to gut leakiness and elevations in serum endotoxin levels (Phelps et al., 2014; Cho et al., 2018). In this sense, our recent study shows that OEA exerts a potent anti-inflammatory action in the gut after alcohol binge drinking, reducing colonic TLR4 expression and oxidative/nitrosative stress. Interestingly, OEA i.p. pre-treatment partially counteracts the alcohol-induced damage to the intestinal barrier, since the integrity of some colonic TJ proteins, such as claudin-3 and occludin, is preserved in OEA-pretreated animals (Antón et al., 2018). In the same study, we did not find an effect of OEA in alcohol-induced gut cell apoptosis, indicative that OEA reduces alcohol-induced paracellular but not transepithelial mechanisms in the colon.

Although the anti-inflammatory and TJ protectant actions of i.p. OEA were clear in a binge alcohol model, reduction of alcohol-induced LPS was partial and modification in alcohol-induced translocation of bacteria was not evident (not significant). This is probably due to the convergence of paracellular and transepithelial mechanisms after an aggressive binge alcohol protocol. However, an oral pretreatment with OEA reduces the bacterial translocation from intestinal lumen to mesenteric lymph nodes and the rise in plasma LPS after ethanol binges, which could be an unquestionable focus for its anti-inflammatory activity (Antón et al., 2018). The differences between i.p. and oral administration modulating paracellular and transepithelial mechanisms in the gut after alcohol administration are unknown and deserve further investigation.

### Microbiome and Gut–Brain Axis Modulation

There is an emerging *hot topic* in the scientific community trying to understand neuropsychological and neuropsychiatric disorders from a holistic perspective, avoiding an exclusive brain-related point or view to clear the way out for a role of the human gut microbiome in the etiology and pathogenesis of such disorders. The influence of the gut microbiota is being explored for many neuropsychiatric conditions and this psychoneuroimmunology approach is extending to the drug addiction field as well. Growing evidence indicates that there is a bidirectional communication between gut microbiota and the brain affecting the CNS functioning with consequences in cognition, emotion, and behavior (reviewed in Sarkar et al., 2018). This gut–brain axis appears to be dysregulated in several brain pathologies, inducing an exacerbated inflammatory response that may ultimately affect the brain. However, we are still far away from the understanding of a role for bacteria signaling, if any, in alcohol responses, including neuroinflammation.

In this regard, very recent studies point out to a role of OEA modulating gut microbiota composition in mice under physiological conditions (Di Paola et al., 2018), polarizing gut-specific immune responses. The study of OEA effects in the gut microbiome altered by alcohol and its relationship with immune and inflammatory responses is still pendent by the scientific community.

## Effects of OEA in Alcohol Consumption and Negative Behaviors Associated With the Abstinence

### OEA Decreases Alcohol Intake and Relapse

**FIGURE 4:**
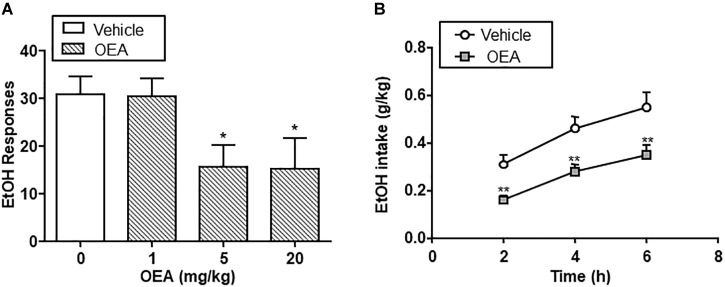
Effect of oleoylethanolamide (OEA) in alcohol self-administration **(A)** and alcohol drinking in a two-bottle choice paradigm **(B)**. Modified from Bilbao et al. (2016).

The beneficial actions of OEA in alcohol abuse have been tested in animal models of chronic alcohol consumption (Bilbao et al., 2016) and binge drinking (Antón et al., 2017a). Regarding chronic alcohol consumption, OEA was able to decrease alcohol self-administration relative to controls at doses of 5 and 20 mg/kg i.p. In a two-bottle choice paradigm, OEA-treated animals showed decreased voluntary drinking relative to controls at different time points, indicating that OEA serves as a regulator of voluntary alcohol intake (Figure 4).

Oleoylethanolamide decreased also alcohol-induced operant responses after a period of drug deprivation, with has important implications for relapse to alcohol. Specifically, OEA was capable of blocking cue-conditioned reinstatement of alcohol-seeking behavior, an animal model of relapse (Bilbao et al., 2016).

### Mechanisms by Which OEA Reduces Alcohol Consumption

It is not clear enough and a focus of debate whether the alcohol-induced neuroinflammation would play a role in alcohol consumption. Some studies fail to detect a link between alcohol-induced neuroinflammation and drug consumption (Lainiola and Linden, 2017), and genetic and pharmacologic manipulation of TLR4 has been shown to have a minimal impact on rodent ethanol consumption (Harris et al., 2017). On the contrary, recent studies using anti-inflammatory mesenchymal stem cells suggest that targeting neuroinflammation could be an effective therapeutic approach to reduce alcohol consumption and relapse (Ezquer et al., 2018, 2017).

In this regard, it is to note that OEA affects also the self-administration of sucrose, so it may affect general motivational processes. Alcohol is both a high-calorie food and a drug of abuse and, therefore, capable of modulating the reward system. In this sense, OEA might serve to modulate motivational drives associated with food selection through an ability to modulate the brain reward system (Tellez et al., 2013). Indeed, it has been described that OEA modulates nicotine-induced activation of mesocorticolimbic dopaminergic neurons (Melis et al., 2008; Mascia et al., 2011). It is also possible that OEA affects cognitive processes associated with the hedonic value of behaviors, since OEA appears to modulate memory consolidation (Campolongo et al., 2009).

Inspite of that, OEA appears to reduce food intake through a peripheral mechanism (Rodriguez de Fonseca et al., 2001) and, similarly, to decrease alcohol intake through activation of the vagus nerve (Bilbao et al., 2016). In detail, Tellez et al. (2013) showed that intestinal OEA modulates vagus nerve terminals in the gut affecting the dopaminergic activity in the reward system and that exogenous administration of OEA restored the dopamine deficiency associated with high-fat diets (Tellez et al., 2013). Regarding alcohol intake, deafferentation of gut vagal afferents with the neurotoxin capsaicin abolished the reduction in alcohol self-administration induced by OEA (Bilbao et al., 2016), suggesting that the modulation of voluntary alcohol consumption by OEA initiates its action in the gut. In favor to this hypothesis is the fact that direct administration of OEA in the brain was not able to alter alcohol self-administration, inspite of OEA levels increase in the brain (nucleus accumbens) after ethanol administration.

The mechanisms by which OEA reduces voluntary alcohol consumption and alcohol relapse have been studied. As mentioned before, OEA is a PPARα agonist and a great deal of its effects are absent in PPARα knock-out mice (Fu et al., 2003; Guzmán et al., 2004). Then, the effects of OEA on alcohol consumption have been compared with the effects of PPARα agonists and antagonists. The i.p. injection of Wy14643, a PPARα agonist, imitated the actions of decreasing alcohol self-administration, cue-induced reinstatement, or alcohol deprivation effect, and the compound GW6471, a PPARα antagonist, reverted the actions of OEA decreasing alcohol intake in the two-bottle choice paradigm. Additionally, deafferentation by capsaicin reverted the actions of Wy14643 on self-administration, similar to the OEA-induced effects.

### OEA Decreases Alcohol Withdrawal-Induced Negative Emotional Behaviors

**FIGURE 5:**
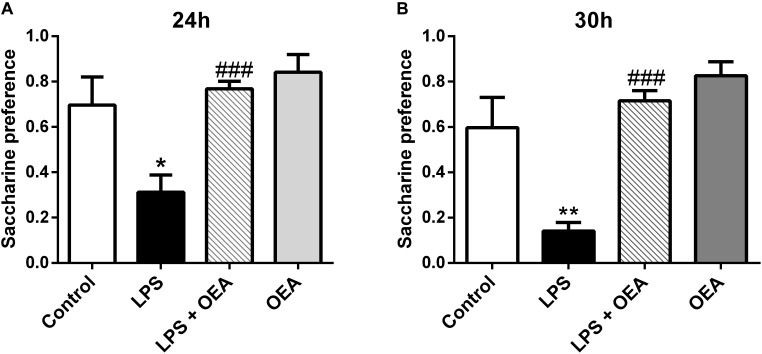
Anti-anhedonic effects of OEA in the saccharine preference test 24 h **(A)** and 30 h **(B)** after LPS administration. Modified from Sayd et al. (2015).

**FIGURE 6:**
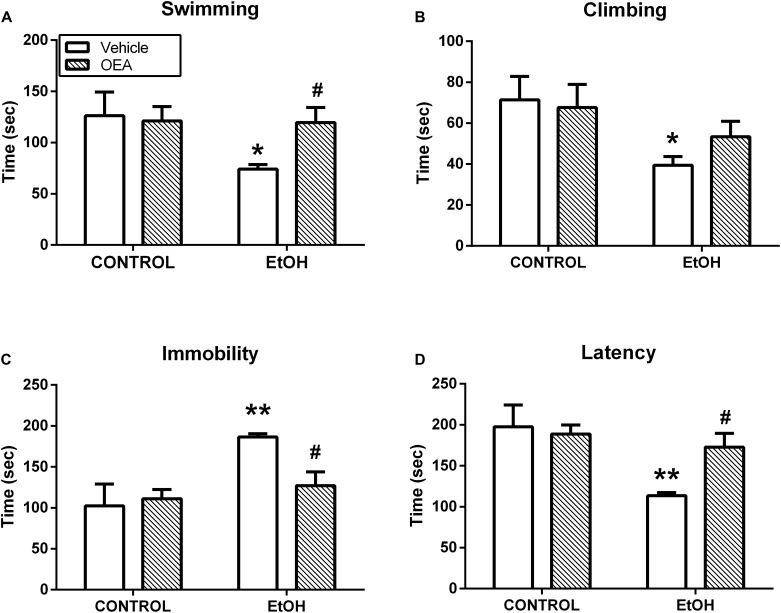
Antidepressant actions of OEA in the forced swimming test after alcohol binges. OEA pretreatment increases the time in which the animals try to escape **(A)** and the latency **(D)** to stay immobile, reducing immobility **(C)**, which is a sign of behavioral despair induced by alcohol binges. OEA did not significantly affect ethanol-induced decrease in climbing **(B)**. Retrieved from Antón et al. (2017a).

Oleoylethanolamide and other acylethanolamides, as mentioned before, display antioxidant and anti-inflammatory properties (Deplanque et al., 2003; Bordet et al., 2006), both of which can also have a profound impact on brain and behavior (Berthold-Losleben et al., 2009; Pandya et al., 2013; Sominsky et al., 2015). For example, inflammatory processes are attributed an important role in the pathophysiology of key manifestations of multiple psychiatric disorders such as anhedonia or deficits in motivation, and those manifestations can be readily induced by the former (Ritsner, 2014; Swardfager et al., 2016). OEA, but not PEA, improves anhedonia in a murine model of neuroinflammation, and this improvement appears to be mediated by its anti-inflammatory properties (Sayd et al., 2015; Figure 5). In this line, OEA induces improvements in depression indexes such as increased swimming time in the behavioral despair test after alcohol binge-induced brain damage and neuroinflammation (Antón et al., 2017a; Figure 6).

Exacerbated neuroinflammation (e.g., by LPS administration) induces sickness behavior, which is evaluated by acute-phase responses, such as activation of HPA axis, body temperature regulation, anhedonia, or behavioral despair. Hyperactivity in the HPA axis has long been linked to anxiety and stress disorders (see Arborelius et al., 1999). OEA administration is able to normalize the hyperactivity of the HPA system in a mice model of chronic mild stress, or after alcohol binge drinking, along with the reversal of behavioral markers of stress such as reduced sucrose drinking or exploratory activity (Jin et al., 2015). The integrity of PPARα and histamine receptors appears to be indispensable for OEA antidepressant effects to take place, as mice deficient for these receptors does not show improvements in mobility time in the tail suspension test or enhanced exploratory time in an open field (Costa et al., 2018).

Hypothermia is an adaptive response to combat acute inflammation and enhance survival (Maes et al., 2012). OEA pre-treatment potentiates the hypothermic acute response after LPS administration and counteracts LPS-induced increases in hypothalamic pyrogen-related molecules such as IL-1β, COX-2, and PGE2. Then, OEA has been proven to modulate acute-phase responses after LPS-induced neuroinflammation, including temperature regulation, HPA axis activation, and motivational arousal (Sayd et al., 2015).

Lipopolysaccharide-induced neuroinflammation is associated also with learning and memory deficits in rodents (Sadraie et al., 2018) and it is well known that the effect of alcohol abuse in memory impairments. OEA appears to have a beneficial role in memory consolidation, through activation of PPARα receptors in the periphery (Campolongo et al., 2009; Provensi et al., 2017). OEA may modulate also emotional memory tasks such as the contextual fear conditioning, which appears very relevant as this behavioral paradigm is generally considered to have substantial coincidences with functional and neural mechanisms involved in anxiety and various other mental disorders (Rosen and Schulkin, 1998; Briscione et al., 2014), including alcohol addiction.

Oleoylethanolamide and other cannabinoid-like compounds have been proposed for the treatment of anxiety disorders and one of the mechanisms by which this compound could have a beneficial impact on anxiety could be slowing down of AEA degradation by competing with it for FAAH activity (Tambaro and Bortolato, 2012). FAAH inhibition has been shown to exert rapid and long-lasting anti-anxiety effects in behavioral paradigms such as acute inescapable stress or chronic corticosterone administration, but the specific mechanisms are still not well understood (Duan et al., 2017). Curiously, FAAH inhibition has also been shown to enhance memory acquisition, possibly through the activation of PPARα receptors (Mazzola et al., 2009). Anyhow, OEA shows clear antidepressant and anti-anxiety properties and this line of intervention is thought to have lower unwanted effects than traditional anti-anxiety drugs (Jin et al., 2015; Duan et al., 2017).

**FIGURE 7:**
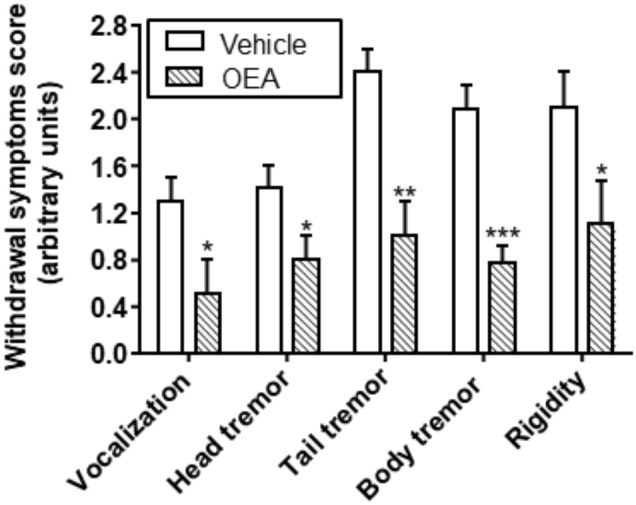
Effect of OEA reducing ethanol withdrawal scores. Retrieved from Bilbao et al. (2016).

Emotional arousal, memory, and learning or motivational behavior are aspects of high relevance in the field of alcohol abuse. OEA modulates also other behaviors related with alcohol withdrawal symptoms, such as vocalization, tremors, or paw rigidity, with a significant reduction in the global ethanol withdrawal scores (Bilbao et al., 2016; Figure 7).

## Conclusion and Clinical Relevance: the Homeostatic Role of OEA in Alcohol Abuse

Data presented in this review indicate that OEA has a beneficial role to counteract many responses related to alcohol consumption and abuse, so OEA-based pharmacotherapies emerge as future perspectives to treat AUDs.

In line with the results presented in this review, OEA can be considered as an endogenous signal with a homeostatic role in the organism. This is supported by the observed increase in OEA levels in the brain and periphery after insults to the CNS that curse with neuroinflammation, and by its anti-inflammatory and protective effects in many CNS disorders, including Parkinson’s disease (Gonzalez-Aparicio et al., 2014), autoimmune encephalomyelitis (Diab et al., 2004), multiple sclerosis (Racke et al., 2006), ischemia (Zhou et al., 2012), anhedonia (Sayd et al., 2015), and cocaine, nicotine, or alcohol addiction (Melis et al., 2008; Bilbao et al., 2013, 2016; Antón et al., 2017a).

In particular, there is strong evidence that endogenous OEA is released as counteracted response to alcohol abuse. First of all, animal studies showed that acute i.p. administrations of ethanol induce a rapid release of OEA (in 45–90 min) in the small intestine, the liver, and also in brain areas such as the nucleus accumbens and cerebellum. Elevations of OEA in plasma after alcohol i.p. administration occurred in an interval of 90–240 min, parallel to the time in which blood alcohol levels are elevated after the administration. Chronic alcohol consumption maintains elevated levels of OEA in plasma during the alcohol liquid diet that remains elevated in the period in which blood ethanol levels are detectable (6 h of withdrawal approximately). Thus, the presence of OEA in blood persisted, at least, as long as the ethanol is also present, and starts to decrease after removing alcohol (Ferrer et al., 2007; Bilbao et al., 2016), strengthening the homeostatic role of OEA in the organism.

Secondly, results in animals are in agreement with recent studies in alcohol-dependent patients. OEA concentration was found elevated during early abstinence in those patients relative to controls, and the concentration of OEA was gradually decreasing as advanced the duration of abstinence, approaching the control group (Garcia-Marchena et al., 2016). Thus, OEA plasma levels were negatively correlated to the duration of abstinence, suggesting that OEA may serve also as a potential marker to predict length of alcohol abstinence.

Thirdly, human studies revealed that OEA is released after different patterns of alcohol or cocaine abuse, and correlates with inflammatory markers. For example, regular alcohol binge drinkers showed during abstinence elevated plasma levels of OEA and other related biolipids, compared with little or no drinkers (Antón et al., 2018). These elevated levels of OEA correlate with key peripheral inflammatory makers found in these young drinkers, such as the innate immunity receptors TLR4, pro-inflammatory cytokines IL-1β and IL-6, or pro-inflammatory enzyme COX-2 (Orio et al., 2017; Antón et al., 2018). Higher plasma HMBG1 and other pro-inflammatory cytokines correlate with worse scores on episodic memory and executive functioning task in female regular binge drinkers and, interestingly, that increase in the danger-related molecule HMGB1 correlates also with OEA plasma levels in such female binge drinkers (Antón et al., 2017b), in favor of the mentioned homeostatic role of OEA.

In summary, preclinical and clinical evidence point-out to the beneficial actions of OEA to treat many negative aspects of alcohol abuse, including neuroinflammation, cognitive decline, withdrawal responses, or motivation and relapse to drinking. OEA-based pharmacotherapies emerge as future perspectives to treat AUDs.

## Author Contributions

LO designed the structure of review and wrote the abstract and sections “Mechanisms by Which OEA Reduces Alcohol-Induced Neuroinflammation” and “Conclusion and Clinical Relevance: the Homeostatic Role of OEA in Alcohol Abuse” of the review. BG-B, FP, AS, and FA wrote sections “Introduction: Conceptualization of Neuroinflammation and Its Relationships With Neuropsychiatric Disorders,” “Alcohol Abuse and Neuroinflammation,” “Oleoylethanolamide Is a Member of the Anti-inflammatory and Neuroprotectant Acylethanolamide Family,” and “Effects of OEA in Alcohol Consumption and Negative Behaviors Associated With Abstinence,” respectively. All authors graphed the figures and revised the final version of the manuscript.

## Conflict of Interest Statement

LO declares to have a licensed patent related to the work. The remaining authors declare that the research was conducted in the absence of any commercial or financial relationships that could be construed as a potential conflict of interest.
